# Immune Regulatory Endotypes Defined by TRIM-Dependent Ubiquitin Signaling and IFN–NF-κB Network Activity in Ankylosing Spondylitis

**DOI:** 10.3390/ijms27135823

**Published:** 2026-06-27

**Authors:** Sevil Ceyhan Dogan, Tugba Agbektas, Mert Atas, Gonca Kabak, Ayca Tas, Yavuz Silig

**Affiliations:** 1 Department of Physical Medicine and Rehabilitation, Faculty of Medicine, Sivas Cumhuriyet University, Sivas 58140, Turkey; drsevilceyhan@gmail.com (S.C.D.); mertatas.18@gmail.com (M.A.); 2 Department of Food Processing Technologies, Yıldızeli Vocational School, Sivas Cumhuriyet University, Sivas 58140, Turkey; tubaagbektas@cumhuriyet.edu.tr; 3 Department of Medical Biochemistry, Faculty of Medicine, Sivas Cumhuriyet University, Sivas 58140, Turkey; gonca.kabak24@gmail.com (G.K.); ysilig@cumhuriyet.edu.tr (Y.S.)

**Keywords:** ankylosing spondylitis, NF-κB signaling, interferon signaling, *TRIM* genes

## Abstract

Ankylosing Spondylitis (AS) is a chronic inflammatory autoimmune rheumatic disease that primarily affects the spine and sacroiliac joints. This study aimed to investigate the expression levels of immune response-related genes, including *IRF7*, *NFKB1A*, *TNFAIP3*, *STAT1*, *TRIM21*, *TRIM22*, and *TRIM25*, as well as the serum levels of CXCL10 and SIRPA proteins in patients with AS. In addition, the potential diagnostic performance of these molecular and serum biomarkers in distinguishing patients with AS from healthy controls was evaluated. A total of 45 patients with AS and 44 healthy controls were included in the study. Immune-related gene expression levels were analyzed using RT-PCR. In addition, serum CXCL10 and SIRPA protein levels were evaluated using ELISA. The expression levels of *NFKB1A*, *TNFAIP3*, *IRF7*, *STAT1*, and *TRIM21* were significantly increased in patients with AS compared to healthy controls (*p* < 0.05). In contrast, no significant differences were detected in the expression levels of *TRIM22* and *TRIM25*. In the ROC analysis, the highest diagnostic performance was obtained for *NFKB1A* (AUC = 0.741), *TNFAIP3* (AUC = 0.720), and *TRIM21* (AUC = 0.722). Serum CXCL10 and SIRPA levels were not significantly different between the groups. In AS, genes particularly associated with NF-κB and interferon signaling pathways (*NFKB1A*, *TNFAIP3*, *IRF7*, *STAT1*, and *TRIM21*) were found to be significantly altered, and these genes may serve as potential molecular biomarkers for AS. In contrast, the diagnostic power of serum protein biomarkers is limited. These findings indicate that the potential of these genes as biomarkers for AS pathogenesis should be further supported by advanced studies evaluating their expression levels.

## 1. Introduction

Ankylosing Spondylitis (AS) is a rheumatic disease characterized by chronic inflammation and new bone formation affecting the axial skeleton [1]. Genetic predisposition (particularly HLA-B27), environmental triggers, and interactions between innate and adaptive immune system components play a role in the pathogenesis of the disease [2]. Recent studies have demonstrated that immune networks involving the coordinated regulation of interferon (IFN) responses and the NF-κB signaling pathway may be determinant factors of disease severity and clinical heterogeneity [3,4]. In this context, ubiquitin-dependent signaling regulation mediated by Tripartite Motif (TRIM) proteins contributes to the identification of novel immune regulatory endotypes in AS [5]. Recent studies have highlighted the importance of interferon-related molecular signatures in AS. In particular, a 2025 study demonstrated that type I interferon-associated pathways contribute to resistance to TNF inhibitor therapy and may define distinct inflammatory endotypes in patients with AS [6]. In addition, multi-omics analyses have revealed that interferon-linked molecular alterations are closely associated with metabolic and immune dysregulation in AS, supporting the concept that coordinated immune signaling networks contribute to disease heterogeneity and progression [7]. These findings emphasize the importance of investigating interferon-regulated genes and NF-κB-associated pathways as potential molecular biomarkers and therapeutic targets for AS.

The TRIM family of proteins plays a critical role in intracellular signal transduction by exhibiting E3 ubiquitin ligase activity [5]. In particular, *TRIM21*, *TRIM22*, and *TRIM25* are closely associated with antiviral responses and type I interferon (IFN) production [8]. These proteins either trigger proteasomal degradation through the ubiquitination of target proteins or modulate the activation of the IFN and NF-κB pathways by altering the stability of signaling complexes [5,8]. For example, *TRIM25* is an important regulator of RIG-I-like receptor signaling and enhances interferon production, whereas *TRIM21* and *TRIM22* may balance the severity of the inflammatory response [5,9].

The IFN–NF-κB axis is a central transcriptional network involved in the pathogenesis of AS [3,4]. Transcription factors, such as *STAT1* and *IRF7*, increase the expression of antiviral and proinflammatory genes following interferon stimulation [3]. *STAT1* is a principal mediator of type I and type II interferon signaling and plays a role in sustaining inflammation [3]. *IRF7* is involved in the amplification of interferon production and is a key determinant of the early phase of the immune response [10]. The regulation of the NF-κB pathway is mediated by negative regulators such as NFKB1A (IκBα) and *TNFAIP3* (A20) [4]. NFKB1A limits activation by retaining NF-κB complexes in the cytoplasm [11]. *TNFAIP3*, on the other hand, is a ubiquitin-regulating enzyme that acts as a potent anti-inflammatory factor by terminating NF-κB signaling [4]. Alterations in the expression or functional impairment of these genes may contribute to persistent chronic inflammation [12].

Proteins such as CXCL10 (IP-10) and SIRPα also play important roles in immune cell communication and migration into tissues [13,14]. CXCL10 is particularly involved in the recruitment of Th1 cells to inflammatory sites, and its elevated levels in patients with AS have been associated with disease activity [13]. SIRPα is an inhibitory receptor expressed on macrophages and dendritic cells that contributes to the regulation of immune responses [14]. The combined evaluation of these molecular components may enable the identification of distinct immune regulatory endotypes in patients with AS [2]. The integration of TRIM-dependent ubiquitin signaling with IFN and NF-κB networks may allow the biological stratification of patients into different subgroups [3,5]. This approach may contribute to a better understanding of disease mechanisms and the development of targeted therapeutic strategies [1]. Although several circulating inflammatory mediators have previously been investigated in AS, CXCL10 and SIRPα were specifically selected in the present study because they represent complementary aspects of immune regulation. CXCL10 is an interferon-inducible chemokine involved in the recruitment of activated T cells and amplification of Th1-mediated inflammatory responses, whereas SIRPα functions as an inhibitory immune checkpoint receptor that regulates macrophage activation, phagocytosis, and innate immune homeostasis. Since the present study focused on IFN- and NF-κB-related immune regulatory networks, evaluating circulating CXCL10 and SIRPα together with the expression of *TRIM21*, *TRIM22*, *TRIM25*, *IRF7*, *STAT1*, *NFKB1A*, and *TNFAIP3* was expected to provide complementary information regarding both transcriptional immune activation and systemic protein level immune regulation in AS.

## 2. Results

### 2.1. Study Population and Baseline Clinical Profile

**Figure 1:**
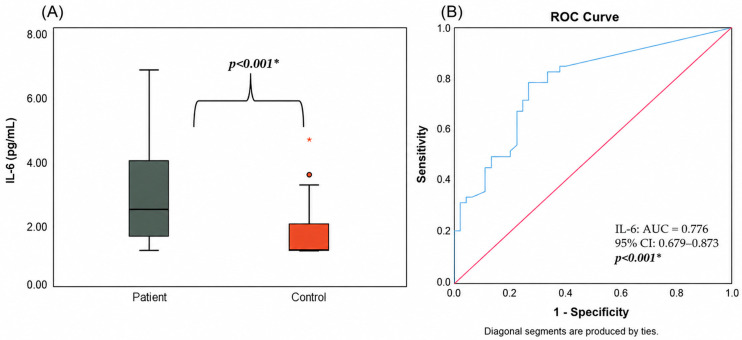
Serum IL-6 levels and diagnostic performance in ankylosing spondylitis. (**A**) Box plot showing serum IL-6 concentrations in patients with AS and healthy controls. IL-6 levels were significantly higher in the patient group than in controls (*p* < 0.001). (**B**) ROC curve analysis demonstrating the diagnostic performance of IL-6 in differentiating AS patients from healthy controls. Significant indicates * *p* < 0.05 (AUC = 0.776, 95% CI: 0.679–0.873, *p* < 0.001).

The baseline categorical characteristics of patients with AS and healthy controls are presented in Table 1. The study population included 45 patients with AS and 44 healthy controls. The sex distribution was similar between the groups, with no statistically significant difference observed between the patients and controls (*p* = 0.444). Among patients with available HLA-B27 data, 26 patients were HLA-B27-positive, corresponding to 68.4% of the evaluated patient subgroup. Regarding treatment status, most patients received anti-TNF therapy, while a smaller proportion were treated with NSAID + DMARD therapy. The continuous demographic, clinical, and inflammatory parameters are presented in Table 1. No significant differences were observed between the patients and controls in terms of age, height, weight, or BMI, indicating that the groups were comparable with respect to demographic and anthropometric characteristics. In contrast, inflammatory markers were significantly elevated in the AS group. CRP levels were markedly higher in patients than in controls (6.71 ± 10.61 vs. 1.39 ± 1.23 mg/L; *p* < 0.001). Similarly, ESR and IL-6 levels were significantly higher in patients than in healthy controls (*p* = 0.016 and *p* < 0.001, respectively; Figure 1). ROC curve analysis further demonstrated that IL-6 had significant diagnostic performance in distinguishing patients with AS from healthy controls (AUC = 0.776, 95% CI: 0.679–0.873, *p* < 0.001; Figure 1). These findings indicate that IL-6 is not only elevated in patients with AS but also shows moderate discriminatory capacity in the study cohort.

### 2.2. Immune-Related Gene Expression Changes in AS

**Figure 2:**
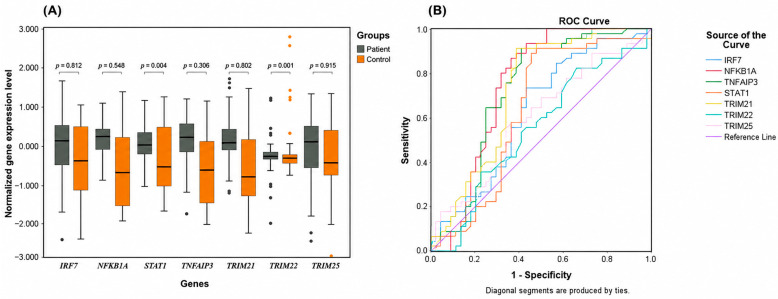
Gene expression profiles and diagnostic performance of immune-related markers in AS. (**A**) Box plots showing the distribution of standardized gene expression values for immune-related markers in AS patients and healthy controls. (**B**) ROC curve analysis demonstrating the diagnostic performance of immune-related gene expression markers in differentiating patients with AS from healthy controls.

The expression levels of the immune-related genes are shown in Table 2 and Figure 2. Several gene expression markers differed significantly between patients with AS and healthy controls. *IRF7* expression was significantly higher in the patient group compared with controls (*p* = 0.041). More pronounced differences were observed for NFKB1A, *TNFAIP3*, and *TRIM21*, all of which were significantly elevated in patients (*p* < 0.001 for each). Moreover, *STAT1* expression was significantly increased in the AS group (*p* = 0.034). In contrast, *TRIM22* and *TRIM25* expression levels did not differ significantly between patients and controls. Overall, these findings suggest that selected immune regulatory genes, particularly NFKB1A, *TNFAIP3*, and *TRIM21*, are more prominently altered in AS than *TRIM22* and *TRIM25*.

Additional subgroup analyses were performed within the AS group according to the HLA-B27 status and treatment category (Table 3). No statistically significant differences were observed in *IRF7, NFKB1A*, *TNFAIP3*, *STAT1*, *TRIM21*, *TRIM22*, *TRIM25*, IL-6, CXCL10, or SIRPA expression levels between HLA-B27-positive and HLA-B27-negative patients (all *p* > 0.05). Similarly, these parameters did not differ significantly between anti-TNF-treated patients and those receiving NSAID + DMARD therapy (all *p* > 0.05). Although *TRIM21* showed a borderline trend according to HLA-B27 status (*p* = 0.062), and NFKB1A showed a borderline trend according to treatment category (*p* = 0.086), these differences did not reach statistical significance. These findings suggest that the observed immune-related molecular and inflammatory alterations were not significantly influenced by HLA-B27 status or treatment category in the present study cohort.

### 2.3. Independent Predictors of Ankylosing Spondylitis in the Multivariable Model

The results of the multivariate logistic regression analysis are presented in Table 4. The model includes inflammatory parameters and selected gene expression markers. Among the evaluated variables, CRP was the only parameter that remained significantly associated with AS in the multivariable model (OR 1.489, 95% CI 1.072–2.068, *p* = 0.018). This finding indicates that higher CRP levels are independently associated with the presence of AS after adjusting for inflammatory and molecular variables. Although IRF7*, NFKB1A*, *TNFAIP3*, *STAT1*, and *TRIM21* showed significant differences in group comparisons, none of these gene expression markers retained independent statistical significance in the logistic regression analysis. Similarly, ESR and IL-6 were not independently associated with disease status after adjusting for the other variables included in the model.

### 2.4. Diagnostic Utility of Gene Expression Biomarkers

The diagnostic performance of the immune-related gene expression markers was evaluated using ROC curve analysis, as shown in Table 5 and Figure 2. Among the analyzed genes*, NFKB1A* demonstrated the highest diagnostic performance for distinguishing patients with AS from healthy controls, with an AUC of 0.741 (95% CI: 0.629–0.853, *p* < 0.001). *TRIM21* and *TNFAIP3* also showed significant discriminatory capacity, with AUC values of 0.722 and 0.720, respectively. IRF7 and *STAT1* demonstrated statistically significant but relatively modest diagnostic performance. In contrast, *TRIM22* and *TRIM25* did not show significant diagnostic value. These results indicate that NFKB1A, *TNFAIP3*, and *TRIM21* may represent the most promising gene expression-based biomarkers among the investigated markers.

### 2.5. Protein Level Analyses

**Figure 3:**
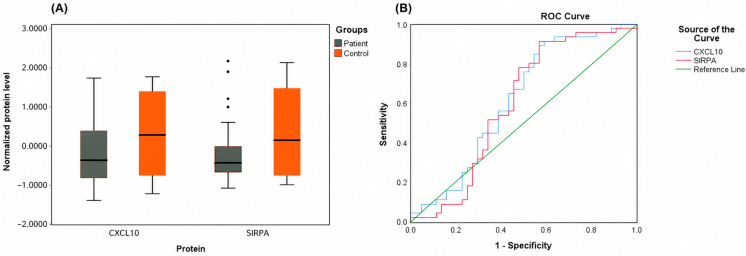
Serum protein biomarker levels and diagnostic performance in AS. (**A**) Box plot showing the distribution of serum CXCL10 and SIRPA standardized protein concentrations in the study cohort. (**B**) ROC curve analysis demonstrating the diagnostic performance of CXCL10 and SIRPA protein biomarkers in differentiating patients with AS from healthy controls.

Serum protein levels of CXCL10 and SIRPA are compared between patients and controls in Table 6 and Figure 3. Both CXCL10 and SIRPA levels were numerically lower in patients with AS than in healthy controls. However, these differences were not statistically significant. CXCL10 showed a borderline trend toward lower levels in the patient group (*p* = 0.061), whereas SIRPA levels were not significantly different between the groups (*p* = 0.117). The diagnostic performances of these protein biomarkers are presented in Table 7. ROC analysis showed a limited discriminatory ability for both CXCL10 and SIRPA. CXCL10 had an AUC of 0.615, and SIRPA had an AUC of 0.596; neither reached statistical significance. These results suggest that, unlike gene expression markers, the evaluated serum protein biomarkers did not provide strong diagnostic discrimination between patients and controls.

### 2.6. Integrated Correlation Analysis of Clinical, Inflammatory, Protein, and Gene Expression Parameters

The correlation matrix of the selected anthropometric, clinical, inflammatory, protein, and gene expression parameters is presented in Table 8. Several clinically relevant correlations were observed. BMI was positively correlated with ESR, IL-6, ASDAS-CRP, and ASDAS-ESR, suggesting an association between body composition and inflammatory disease activity. CRP showed a strong positive correlation with IL-6 and a significant positive correlation with ESR and ASDAS-CRP, supporting the relationship between systemic inflammation and disease activity. The disease activity measures were strongly interrelated. The VAS showed strong positive correlations with BASDAI, ASDAS-CRP, and ASDAS-ESR. Similarly, BASDAI was strongly correlated with both ASDAS-CRP and ASDAS-ESR, indicating consistency between patient-reported and composite disease activity indices. At the molecular level, strong positive correlations were observed between several immune-related gene expression markers. *IRF7* was positively correlated with NFKB1A, *TNFAIP3*, *STAT1*, and *TRIM21*. NFKB1A showed particularly strong correlations with *TNFAIP3* and *TRIM21*, whereas *TNFAIP3* was also positively correlated with *STAT1* and *TRIM21*. These findings suggest coordinated expression patterns among immunoregulatory genes in patients with AS. Protein-related correlations were also observed in this study. CXCL10 showed a strong positive correlation with SIRPA, indicating a close relationship between these two serum protein markers. In addition, SIRPA was negatively correlated with NFKB1A and *TNFAIP3*, suggesting that lower SIRPA levels may be associated with the increased expression of selected immune-related genes.

### 2.7. Comparative Interpretation of Gene and Protein Biomarkers

When gene expression and protein biomarkers were evaluated together, the gene expression markers showed more pronounced disease-related alterations than the serum protein markers. *IRF7, NFKB1A*, *TNFAIP3*, *STAT1*, and *TRIM21* were significantly elevated in patients with AS, and several of these genes demonstrated significant diagnostic performance in ROC analysis. In contrast, CXCL10 and SIRPA levels did not significantly differ between patients and controls and showed limited diagnostic value. Overall, these findings suggest that immune-related gene expression changes may better reflect the molecular inflammatory profile of AS than the measured serum protein biomarkers. However, the significant correlations between SIRPA and selected gene expression markers indicate that protein-level changes may still be linked to the broader immune regulatory network involved in the disease.

## 3. Discussion

AS is a complex inflammatory disease that arises from the interaction of genetic predisposition, environmental triggers, and multiple immune signaling pathways. The current literature indicates that TNF-α, the IL-23/IL-17 signaling pathway, and the NF-κB signaling pathway play central roles in the immunopathogenesis of this disease [15]. Consistent with these fundamental mechanisms, the findings of the present study support the notion that NF-κB- and interferon-related gene expression alterations play important roles in AS pathogenesis. The NF-κB signaling pathway is a critical transcriptional network involved in the regulation of pro-inflammatory responses and is considered one of the major mechanisms responsible for sustaining chronic inflammation in patients with AS. In the current study, the increased expression levels of *NFKB1A* and *TNFAIP3* suggest that, together with NF-κB pathway activation, negative feedback mechanisms may also be involved. *TNFAIP3* (A20) is an important regulator that suppresses NF-κB activation and is regarded as a compensatory response aimed at maintaining homeostasis during chronic inflammation [16]. This finding indicates that the inflammatory response in AS progresses not only through activation mechanisms but also through regulatory pathways.

Alterations were also observed in the expression of *IRF7* and *STAT1*, which are associated with the interferon signaling pathways. *IRF7* and *STAT1* are key regulators of the type I interferon response and play critical roles in the antiviral components of innate immunity. The literature suggests that AS may be shaped not only by classical inflammatory cytokines but also by interferon (IFN) signatures [17]. In this context, the present findings imply that systemic immune activation observed in the disease includes interferon-mediated component. The TRIM protein family is an important regulator of innate immune response. *TRIM21* is an interferon-induced E3 ubiquitin ligase involved in immune regulation. Increased *TRIM21* expression supports the activation of cross-regulatory mechanisms between the interferon and NF-κB pathways [18]. In contrast, the absence of significant alterations in *TRIM22* and *TRIM25* expression suggests that members of the TRIM family do not play homogeneous roles in AS pathogenesis and that gene-specific effects may exist.

The IL-23/IL-17 pathway also plays a critical role in AS pathogenesis by directing inflammation and structural alterations through Th17 cell responses [19,20]. The interaction of the NF-κB and JAK/STAT pathways with these immune pathways indicates that the gene expression changes observed in this study were part of a broader inflammatory network. Furthermore, non-coding RNAs have been reported to contribute to disease progression by modulating these signaling pathways [21].

In terms of serum protein biomarkers, previous studies have demonstrated that cytokine levels may be associated with disease activity; however, these biomarkers are known to be significantly influenced by treatment status and interindividual variability [22]. In the present study, while serum protein levels showed limited diagnostic value, gene expression profiles revealed more pronounced differences, suggesting that transcriptomic alterations may represent earlier and more sensitive molecular reflections of the disease. An important consideration when interpreting the present findings is the potential influence of anti-TNF therapy on both transcriptomic and circulating protein biomarkers. Tumor necrosis factor inhibitors are known to modulate inflammatory signaling pathways, including NF-κB activation and interferon-related responses, and may therefore affect the expression of immune-regulatory genes as well as serum cytokine and chemokine levels. Because most patients in our cohort were receiving anti-TNF therapy, treatment-related effects cannot be completely excluded. However, subgroup analyses performed according to treatment category did not reveal significant differences in the expression levels of the investigated genes or serum protein biomarkers between patients receiving anti-TNF therapy and those treated with NSAID plus DMARD therapy. Nevertheless, the relatively small number of patients in the non-biologic treatment subgroup may have limited the statistical power to detect subtle treatment-related differences. Therefore, the observed molecular alterations should be interpreted with caution, and future studies including treatment-naïve patients and longitudinal follow-up are warranted to better distinguish disease-related molecular signatures from therapy-induced changes. Correlation analyses further demonstrated significant positive correlations among immune-related gene expression levels, ranging from weak to strong. This finding supports the concept that inflammatory signaling pathways in AS do not function as isolated entities but as an integrated biological network. The literature also emphasizes that the NF-κB, interferon, and IL-17 pathways interact closely with one another and collectively shape both the inflammatory and structural components of the disease [23].

## 4. Materials and Methods

### 4.1. Patients and Control Group

This case–control study was conducted at the Department of Physical Medicine and Rehabilitation, Sivas Cumhuriyet University Research and Application Hospital. Patient recruitment was carried out between April 2026 and June 2026. Individuals diagnosed with ankylosing spondylitis (AS) according to the Modified New York Criteria proposed by van der Linden et al. were considered eligible for participation [24]. The inclusion criteria for the AS group were as follows: (i) diagnosis of AS according to the Modified New York Criteria, (ii) age ≥ 18 years, (iii) willingness to participate in the study, and (iv) sufficient cognitive ability to complete the clinical questionnaires and assessment scales. The exclusion criteria were: (i) presence of other rheumatic diseases (including rheumatoid arthritis, systemic lupus erythematosus, and scleroderma), (ii) fibromyalgia, (iii) chronic kidney disease, (iv) diabetes mellitus, (v) acute infection, (vi) pregnancy or lactation, and (vii) any history of malignancy. The healthy control group consisted of age- and sex-matched volunteers without a previous diagnosis of ankylosing spondylitis or any chronic inflammatory, autoimmune, or rheumatic disease. The same exclusion criteria applied to the patient group were also applied to the control group. The age range of the participants was 22–65 years in the AS group and 22–64 years in the control group. Disease activity and pain severity were assessed using the Bath Ankylosing Spondylitis Disease Activity Index (BASDAI) [25], Ankylosing Spondylitis Disease Activity Score (ASDAS-CRP and ASDAS-ESR) [26], and Visual Analog Scale (VAS) [27].

The sample size was calculated using G*Power software version 3.1.9.4 (Heinrich-Heine-University, Düsseldorf, Germany). An a priori power analysis for an independent-samples *t*-test was performed assuming an effect size (d) of 0.61, significance level (α) of 0.05, statistical power (1 − β) of 0.81, and allocation ratio (N2/N1) of 1. The analysis indicated that a minimum total sample size of 89 participants was required. The calculated degrees of freedom (df), critical t-value, and noncentrality parameter (δ) were 86, 1.9872899, and 2.8360499, respectively, with an achieved statistical power of 0.8246969. Written informed consent was obtained from all participants prior to enrollment. The study protocol was approved by the Sivas Cumhuriyet University Faculty of Medicine Ethics Committee (Approval Date: 4 April 2026; Approval No: 2026-04/79) and was conducted in accordance with the principles of the Declaration of Helsinki.

### 4.2. Determining Protein Levels

#### 4.2.1. Serum Isolation from Blood Samples

A total of 8 mL of peripheral venous blood was collected from each participant in biochemistry tubes. Blood samples were centrifuged at 3000 rpm for 10 min to separate the serum. The obtained serum samples were stored at −80 °C under appropriate conditions until further analysis.

#### 4.2.2. Protein Levels

CXCL10 and SIRPα protein levels in serum samples were determined using enzyme-linked immunosorbent assay (ELISA). Analyses were performed on 96-well microplates in accordance with the manufacturer’s protocols, using commercially available ELISA kits specific to the relevant proteins. Human CXCL10 ELISA Kit (Cat. No: E3800Hu, Bioassay Technology Laboratory (BT LAB), Shanghai, China) was used for the determination of CXCL10 levels; the kit had a standard curve range of 2–600 ng/L and a sensitivity of 1.22 ng/L. Human SIRPα ELISA Kit (Cat. No: E6367Hu, Bioassay Technology Laboratory (BT LAB), Shanghai, China) was used to determine the SIRPα levels; the kit had a standard curve range of 0.05–20 ng/mL and a sensitivity of 0.031 ng/mL.

In the ELISA procedure, 40 µL of sample dilution buffer and 10 µL of sample were added to each well. The plates were incubated at 37 °C for 30 min. Following incubation, the wells were washed five times with washing buffer, and an HRP-conjugate reagent was added to each well. The plates were then incubated at 37 °C for an additional 30 min. Subsequently, chromogen solutions were added and incubated for 15 min. The reaction was terminated by the addition of a stop solution, and the resulting color change was measured spectrophotometrically at 450 nm. Using the obtained absorbance values, CXCL10 and SIRPα protein levels were quantitatively calculated based on a standard curve.

### 4.3. Determining Gene Expression Levels

#### 4.3.1. RNA Isolation

To determine the expression levels of *TRIM21*, *TRIM22*, *TRIM25*, *STAT1*, *IRF7, NFKB1A*, and *TNFAIP3*, 4 mL of peripheral venous blood was collected in EDTA-containing tubes from individuals diagnosed with AS and healthy control subjects. Total RNA was isolated from the obtained blood samples in accordance with the protocol provided by the manufacturer of the commercial RNA isolation kit. Following isolation, the quantity and purity of the RNA samples were evaluated spectrophotometrically using a NanoDrop spectrophotometer (Thermo Fisher Scientific, Waltham, MA, USA), and samples with an A260/A280 ratio between 1.8 and 2.0 were included in the analyses. No-template controls were included in every RT-qPCR run to exclude reagent contamination. Melt curve analysis was performed after amplification to verify amplification specificity and the absence of primer-dimer formation. All reactions were performed in duplicate, and only reproducible Ct values were included in the final analyses. RNA samples that met the purity and quality control criteria were stored at −80 °C until further analysis.

#### 4.3.2. cDNA Synthesis

Complementary DNA (cDNA) synthesis was performed according to the manufacturer’s instructions (A.B.T., Berlin, Germany) using the relevant kit protocol. Briefly, cDNA was synthesized using a specified amount of total RNA. The obtained cDNA samples were stored at −80 °C for subsequent gene expression analyses.

#### 4.3.3. Gene Expression Levels

Following cDNA synthesis, the gene expression levels of *TRIM21*, *TRIM22*, *TRIM25*, *STAT1*, *IRF7, NFKB1A*, and *TNFAIP3* were analyzed in the obtained samples. For this purpose, real-time polymerase chain reaction (RT-qPCR) was performed using optimized gene-specific primers for each target gene. The primer sequences were proprietary and supplied by the manufacturer (AXACELL Biosassay, Türkiye). Optimized primer sets specific to each target gene were used according to the manufacturer’s recommendations. The corresponding catalog numbers are provided in Table 9. According to the manufacturer’s validation data, all primer sets exhibited amplification efficiencies within the acceptable range (90–110%) for quantitative gene expression analysis.

SYBR Green (Cat No: GK10002, GipBio, Shanghai, China) fluorescent dye was used for gene expression analysis, and the amplification process was performed according to the manufacturer’s protocol (Table 10 and Table 11). The obtained Ct values were normalized using GAPDH as the reference gene, and changes in gene expression levels were calculated using the ΔΔCt (Delta Ct) method. Using this approach, the relative differences in gene expression between individuals with AS and healthy controls were comparatively evaluated. Gene expression data were analyzed using the ΔΔCt method through the Qiagen Data Analysis Center, and the obtained results were subsequently evaluated using the Rotor-Gene 6000 and RT^2^ Profiler qPCR Array Data Analysis Software (version 3.5).

#### 4.3.4. Statistical Analysis

All statistical analyses were performed using SPSS software version 23.0 (IBM Corp., Armonk, NY, USA). Graphical representations, including box plots, were prepared using GraphPad Prism version 8.0.1 (GraphPad Software, San Diego, CA, USA). Prior to the comparative analyses, the distribution pattern of the numerical variables was evaluated using the Shapiro–Wilk normality test. Continuous data were summarized as mean ± standard deviation, together with minimum and maximum values. For variables that did not show a normal distribution, median values were considered during interpretation. Comparisons between patients with AS and healthy controls were performed using the Mann–Whitney U test or independent samples Student’s *t*-test, depending on the distributional properties of the data. In particular, immune-related gene expression parameters were compared between the case and control groups using Student’s *t*-test, where appropriate. Categorical variables were expressed as numbers and percentages, and intergroup differences were analyzed using the chi-square test for independence. To identify factors associated with AS, multivariable logistic regression analysis was performed, including inflammatory markers and selected gene expression parameters. The results are presented as regression coefficients, standard errors, odds ratios [Exp(B)], and 95% confidence intervals. The diagnostic performance of the gene expression markers and serum protein levels was evaluated using receiver operating characteristic (ROC) curve analysis. The area under the curve (AUC), 95% confidence interval, optimal cutoff value, sensitivity, specificity, positive predictive value, and negative predictive value were calculated. The Youden index was used to determine the most appropriate cutoff points. The positive and negative predictive values were calculated using the sensitivity and specificity values obtained at the selected ROC-derived cut-off points, together with the disease prevalence in the present study cohort. Because the positive and negative predictive values are prevalence-dependent measures, they were interpreted as cohort-specific estimates rather than generalizable population-level diagnostic values. The relationships among anthropometric measurements, inflammatory markers, disease activity scores, serum protein levels, and gene expression parameters were examined using correlation analysis. Correlation coefficients were reported along with their statistical significance levels. A *p*-value < 0.05 was considered statistically significant for all analyses.

## 5. Conclusions

In conclusion, this study demonstrated that immune regulatory genes associated with interferon signaling and the NF-κB pathway, particularly *IRF7, NFKB1A*, *TNFAIP3*, *STAT1*, and *TRIM21*, were significantly altered in patients with AS. These findings suggest that AS is associated with coordinated inflammatory and immune regulatory changes involving interferon-mediated responses, NF-κB-related signaling, and tripartite motif (TRIM)-dependent ubiquitin pathways. Among the investigated markers*, NFKB1A*, *TNFAIP3*, and *TRIM21* showed the strongest diagnostic performance in ROC analyses and may have potential diagnostic relevance in HNSCC. In contrast, serum CXCL10 and SIRPA protein levels had limited diagnostic value. Overall, immune-related gene expression profiles may reflect the inflammatory profile of AS and contribute to the identification of molecular endotypes. However, their biomarker utility and functional relevance should be interpreted cautiously and validated in larger independent multicenter cohorts, supported by transcriptomic, proteomic, and functional analyses.

## 6. Limitations

One of the main limitations of this study is the relatively small sample size and single-center study design, which may limit the generalizability of the findings to a broader patient population. Another important limitation is the absence of a disease control group, such as patients with other inflammatory rheumatic disorders. Therefore, although the investigated markers distinguished patients with AS from healthy controls, their disease specificity should be interpreted with caution. In addition, most patients received anti-TNF therapy, and treatment-related effects may have influenced both the gene expression profiles and serum biomarker levels. Although no significant differences were observed in the gene expression profiles between anti-TNF-treated patients and those receiving NSAID + DMARD therapy, this finding should be interpreted cautiously because of the limited sample size of the NSAID + DMARD subgroup. Another limitation is that the study evaluated gene expression changes only at the transcriptomic level, and complementary protein-level validation analyses were not performed for the investigated genes. Furthermore, the cross-sectional design of the study did not allow for the evaluation of longitudinal changes in gene expression over time, according to disease activity or treatment response. Finally, although significant associations were identified among several immune-related genes, the underlying molecular interactions and biological pathways were not investigated using functional experimental models. Therefore, larger multicenter studies including disease control groups and integrating transcriptomic, proteomic, and functional analyses, are required to validate and extend these findings.

## Figures and Tables

**Table 1:** Demographic, clinical, and inflammatory characteristics of patients with AS and healthy controls.

Parameter	AS Patients (*n* = 45)	Healthy Controls (*n* = 44)	Min	Max	*p* Value
Male, *n* (%)	24 (47.1%)	27 (52.9%)	22.00	65.00	0.444 ^a^
Female, *n* (%)	21 (55.3%)	17 (44.7%)	22.00	64.00	
Age (years)	41.53 ± 11.60	43.14 ± 10.49	147.00	187.00	0.608 ^b^
Height (cm)	167.53 ± 10.07	169.84 ± 8.86	153.00	190.00	0.308 ^b^
Weight (kg)	81.13 ± 15.28	77.73 ± 11.35	52.00	117.00	0.366 ^b^
BMI (kg/m^2^)	29.11 ± 6.14	26.94 ± 3.41	56.00	100.00	0.102 ^b^
Disease duration (years)	11.47 ± 6.93	N/A	19.50	46.87	N/A
Diagnosis duration (years)	8.11 ± 4.90	N/A	20.57	35.76	N/A
HLA-B27-positive, *n* (%)	26/38 (68.4%)	N/A	1.00	25.00	N/A
Anti-TNF treatment, *n* (%)	39/45 (86.7%)	N/A	1.00	22.00	N/A
NSAID + DMARD treatment, *n* (%)	6/45 (13.3%)	N/A	0.170	68.990	N/A
CRP (mg/L)	6.71 ± 10.61	1.39 ± 1.23	0.339	5.290	<0.001 ^b^*
ESR (mm/h)	10.04 ± 7.20	7.20 ± 6.00	2.00	39.00	0.016 ^b^*
IL-6 (pg/mL)	3.48 ± 2.22	1.99 ± 0.83	2.00	23.00	<0.001 ^b^*
VAS	6.64 ± 2.34	N/A	1.50	13.60	N/A
BASDAI	4.80 ± 1.93	N/A	1.50	4.92	N/A
ASDAS-CRP	2.78 ± 0.95	N/A	1.00	10.00	N/A
ASDAS-ESR	2.65 ± 0.79	N/A	0.00	8.90	N/A

^a^ Chi-square test. ^b^ Mann–Whitney U test. HLA-B27 percentages were calculated among patients with available HLA-B27 data (*n* = 38). Treatment percentages were calculated among patients with available treatment data (*n* = 45). N/A: not applicable. * Statistically significant at *p <* 0.05.

**Table 2:** Comparative analysis of immune-related gene expression profiles in patients with AS and healthy controls.

Parameter	Group	Mean ± SD	Min	Max	Median (50th)	*p* Value
*IRF7*	Case	29.98 ± 1.96	23.92	34.14	30.26	**0.041** **^ a^** *
	Control	28.90 ± 2.38	23.88	32.56	28.94	
*NFKB1A*	Case	30.80 ± 1.69	26.94	33.97	30.96	**<0.001** **^ a^** *
	Control	28.03 ± 3.64	23.32	35.02	27.63	
*TNFAIP3*	Case	33.03 ± 1.74	27.60	35.97	33.20	**<0.001** **^ a^** *
	Control	30.97 ± 2.77	26.84	35.82	30.79	
*STAT1*	Case	31.54 ± 2.90	20.27	35.54	31.83	**0.034** **^ a^** *
	Control	30.71 ± 2.78	26.36	35.87	30.01	
*TRIM21*	Case	31.96 ± 1.60	28.64	35.76	31.76	**<0.001** **^ a^** *
	Control	30.24 ± 2.31	26.16	35.14	29.62	
*TRIM22*	Case	26.60 ± 0.86	23.74	29.04	26.56	0.436 ^a^
	Control	26.83 ± 1.91	19.55	32.68	26.51	
*TRIM25*	Case	32.79 ± 2.08	27.22	35.97	33.14	0.111 ^a^
	Control	32.13 ± 2.02	26.31	36.00	31.87	

^a^ Mann–Whitney U test with mean ± standard deviation, minimum–maximum values, and median. * Statistically significant at *p* < 0.05.

**Table 3:** Subgroup analysis of immune-related gene expression, inflammatory, and serum protein markers according to HLA-B27 status and treatment category in patients with AS.

Parameter	HLA-B27-Positive Mean ± SD	HLA-B27-Negative Mean ± SD	*p* Value	Anti-TNF Mean ± SD	NSAID + DMARD Mean ± SD	*p* Value
*IRF7*	30.01 ± 2.11	30.61 ± 1.33	0.423	29.98 ± 1.74	29.92 ± 3.25	0.394
*NFKB1A*	30.79 ± 1.79	31.18 ± 1.83	0.362	30.62 ± 1.65	31.98 ± 1.62	0.086
*TNFAIP3*	33.33 ± 1.49	32.76 ± 2.19	0.638	32.87 ± 1.72	34.08 ± 1.66	0.113
*STAT1*	32.04 ± 1.61	32.07 ± 1.80	0.718	31.32 ± 2.97	33.00 ± 2.02	0.182
*TRIM21*	31.75 ± 1.51	32.53 ± 1.32	0.062	32.04 ± 1.58	31.48 ± 1.77	0.689
*TRIM22*	26.49 ± 0.78	26.37 ± 0.52	0.432	26.66 ± 0.79	26.26 ± 1.29	0.854
*TRIM25*	33.03 ± 1.96	32.87 ± 1.69	0.593	32.97 ± 1.98	31.64 ± 2.57	0.193
IL-6	3.60 ± 2.65	2.96 ± 1.13	0.912	3.47 ± 2.28	3.56 ± 1.96	0.688
CXCL10	4.73 ± 1.20	4.98 ± 1.52	0.826	4.82 ± 1.32	4.75 ± 1.22	0.920
SIRPA	61.24 ± 40.42	68.00 ± 39.14	0.510	63.27 ± 41.49	64.08 ± 27.36	0.841

Values are presented as mean ± standard deviation. Comparisons were performed using the Mann–Whitney U test. HLA-B27 subgroup analysis was performed among patients with available HLA-B27 data. Treatment subgroup analysis was performed between anti-TNF-treated patients and patients receiving NSAID + DMARD therapy. A *p* value < 0.05 was considered statistically significant.

**Table 4:** Multivariable logistic regression analysis of clinical and molecular variables associated with AS.

Variable	B	S.E.	*p* Value	OR [Exp(B)]	95% CI for OR
CRP	0.398	0.168	0.018 *	1.489	1.072–2.068
ESR	−0.040	0.059	0.495	0.960	0.855–1.079
IL-6	0.415	0.300	0.166	1.515	0.841–2.728
*IRF7*	−0.076	0.188	0.689	0.927	0.641–1.341
NFKB1A	0.111	0.198	0.575	1.118	0.758–1.648
*TNFAIP3*	0.298	0.244	0.221	1.348	0.835–2.174
*STAT1*	−0.219	0.195	0.261	0.803	0.548–1.177
*TRIM21*	0.234	0.233	0.315	1.264	0.801–1.995

Values are presented as regression coefficient (B), standard error (S.E.), odds ratio [OR = Exp(B)], and 95% confidence interval. In the binary logistic regression model, healthy controls were coded as 0 and patients with AS were coded as 1. OR: odds ratio; CI: confidence interval; CRP: C-reactive protein; ESR: erythrocyte sedimentation rate; IL-6: interleukin-6. * Statistically significant at *p* < 0.05.

**Table 5:** Diagnostic performance of immune-related gene expression markers for differentiating AS patients from healthy controls.

	*IRF7*	*NFKB1A*	*TNFAIP3*	*STAT1*	*TRIM21*	*TRIM22*	*TRIM25*
**AUC (95% CI)**	0.626 (0.508–0.744)	0.741 (0.629–0.853)	0.720 (0.607–0.833)	0.630 (0.507–0.754)	0.722 (0.612–0.832)	0.548 (0.426–0.670)	0.598 (0.480–0.716)
***p* value**	0.041 *	<0.001 *	<0.001 *	0.034 *	<0.001 *	0.436	0.111
**Cut-off point**	≥29.27	≥28.90	≥31.06	≥30.06	≥30.22	≥26.36	≥32.81
**Sensitivity (%)**	73.3	88.9	91.1	91.1	91.1	82.2	55.6
**Specificity (%)**	56.8	61.4	59.1	52.3	61.4	34.1	65.9
**PPV (%)**	63.4	70.2	69.5	66.1	70.7	56.1	62.5
**NPV (%)**	67.5	84.4	86.7	85.2	87.1	65.2	59.2

* Statistically significant at *p* < 0.05. AUC: area under the ROC curve; CI: confidence interval; PPV: positive predictive value; NPV: negative predictive value.

**Table 6:** Comparative analysis of serum protein levels in patients with AS and healthy controls.

Parameter	Group	Mean ± SD	Min	Max	Median (50th)	*p* Value
CXCL10 (ng/mL)	Case	4.71 ± 1.26	2.88	7.77	4.31	0.061 ^a^
	Control	5.51 ± 1.73	3.11	7.86	5.42	
SIRPA (ng/mL)	Case	61.08 ± 37.68	16.44	189.01	46.42	0.117 ^a^
	Control	89.02 ± 58.46	20.26	187.18	79.19	

^a^ Mann–Whitney U test with mean ± standard deviation, minimum–maximum values, and median. Statistically significant at *p* < 0.05.

**Table 7:** ROC curve analysis demonstrating the diagnostic performance of serum protein markers in AS.

	CXCL10 (ng/mL)	SIRPA (ng/mL)
**AUC (95% CI)**	0.615 (0.495–0.735)	0.596 (0.472–0.720)
***p* value**	0.061	0.117
**Cut-off point**	≤6.40	≤102.81
**Sensitivity (%)**	88.9	91.1
**Specificity (%)**	43.2	43.2
**PPV (%)**	61.5	62.1
**NPV (%)**	79.2	82.6

Statistically significant at *p* < 0.05. AUC: area under the ROC curve; CI: confidence interval; PPV: positive predictive value; NPV: negative predictive value. PPV and NPV were calculated according to the disease prevalence in the present study cohort and should be interpreted as cohort-specific estimates.

**Table 8:** Correlation matrix of selected anthropometric, clinical, inflammatory, protein, and gene expression parameters in the ankylosing spondylitis cohort.

Variable Pair	r	*p* Value
Age–Disease duration	0.607 **	**<0.001**
Age–Diagnosis duration	0.380 *	**0.010**
Disease duration–Diagnosis duration	0.794 **	**<0.001**
BMI–ESR	0.263 *	**0.013**
BMI–IL-6	0.243 *	**0.022**
BMI–ASDAS-CRP	0.377 *	**0.011**
BMI–ASDAS-ESR	0.347 *	**0.020**
CRP–ESR	0.375 **	**<0.001**
CRP–IL-6	0.734 **	**<0.001**
CRP–ASDAS-CRP	0.464 **	**0.001**
ESR–IL-6	0.426 **	**<0.001**
ESR–ASDAS-CRP	0.346 *	**0.020**
ESR–ASDAS-ESR	0.464 **	**0.001**
IL-6–ASDAS-CRP	0.363 *	**0.014**
VAS–BASDAI	0.868 **	**<0.001**
VAS–ASDAS-CRP	0.746 **	**<0.001**
VAS–ASDAS-ESR	0.855 **	**<0.001**
BASDAI–ASDAS-CRP	0.776 **	**<0.001**
BASDAI–ASDAS-ESR	0.867 **	**<0.001**
ASDAS-CRP–ASDAS-ESR	0.842 **	**<0.001**
*IRF7*–*NFKB1A*	0.596 **	**<0.001**
*IRF7*–*TNFAIP3*	0.571 **	**<0.001**
*IRF7*–*STAT1*	0.562 **	**<0.001**
*IRF7*–*TRIM21*	0.620 **	**<0.001**
*IRF7*–*TRIM25*	0.294 **	**0.005**
NFKB1A–*TNFAIP3*	0.863 **	**<0.001**
NFKB1A–*STAT1*	0.598 **	**<0.001**
NFKB1A–*TRIM21*	0.709 **	**<0.001**
*TNFAIP3*–*STAT1*	0.628 **	**<0.001**
*TNFAIP3*–*TRIM21*	0.623 **	**<0.001**
*TNFAIP3*–*TRIM25*	0.246 *	**0.019**
*STAT1*–*TRIM21*	0.566 **	**<0.001**
*STAT1*–*TRIM25*	0.418 **	**<0.001**
*TRIM21*–*TRIM25*	0.301 **	**0.004**
*TRIM22*–*TRIM25*	0.255 *	**0.015**

Values are presented as Pearson correlation coefficients (r). Only statistically significant correlations are shown. * p < 0.05; ** p < 0.01. BMI: body mass index; CRP: C-reactive protein; ESR: erythrocyte sedimentation rate; IL-6: Interleukin-6; VAS: Visual Analog Scale; BASDAI: Bath AS Disease Activity Index; ASDAS-CRP: AS Disease Activity Score based on CRP; ASDAS-ESR: AS Disease Activity Score based on ESR.

**Table 9:** Catalog numbers of optimized primers.

PRT-0405-HU	Interferon Regulatory Factor 7 (*IRF7*) (HUMAN)
PRT-0501-HU	NFKB Inhibitor Alpha (NFKB1A) (HUMAN)
PRT-0953-HU	TNF Alpha-Induced Protein 3 (*TNFAIP3*) (HUMAN)
PRT-0365-HU	Signal Transducer And Activator of Transcription 1 (*STAT1*) (HUMAN)
PRT-0199-HU	Tripartite Motif-Containing 21 (*TRIM21*) (HUMAN)
PRT-0871-HU	Tripartite Motif-Containing 22 (*TRIM22*) (HUMAN)
PRT-0221-HU	Tripartite Motif-Containing 25 (*TRIM25*) (HUMAN)
PRT-0001-HU	Glyceraldehyde 3-phosphate dehydrogenase (GAPDH) (HUMAN)

**Table 10:** RT-qPCR Thermal Cycle Conditions and Reaction Mixture Composition.

Step	Temperature	Time	Cycles
Initial denaturation	95 °C	5 min	1
Denaturation	95 °C	15 s	40 *
Annealing	60 °C	30 s	
Melt curve analysis	65–95 °C	Continuous	1

* The total number of amplification cycles should be adjusted according to the experimental protocol (commonly 40 cycles).

**Table 11:** Components and Volumes Used for RT-qPCR Amplification.

Component	Volume (µL)
2× SYBR Green Master Mix	5.0
Forward primer	0.4
Reverse primer	0.4
Nuclease-free water	3.2
cDNA template	1.0
Total volume	10.0

## Data Availability

The original contributions presented in this study are included in the article. Further inquiries can be directed to the corresponding author.

## Abbreviations

The following abbreviations are used in this manuscript:

Abbreviation	Definition
AS	Ankylosing Spondylitis
AUC	Area Under the Curve
BASDAI	Bath Ankylosing Spondylitis Disease Activity Index
BMI	Body Mass Index
CRP	C-Reactive Protein
CT	Cycle Threshold
CXCL10	C-X-C Motif Chemokine Ligand 10
ELISA	Enzyme-Linked Immunosorbent Assay
ESR	Erythrocyte Sedimentation Rate
GAPDH	Glyceraldehyde-3-Phosphate Dehydrogenase
HLA-B27	Human Leukocyte Antigen B27
IFN	Interferon
IL-6	Interleukin-6
*IRF7*	Interferon Regulatory Factor 7
JAK/STAT	Janus Kinase/Signal Transducer and Activator of Transcription
NF-κB	Nuclear Factor Kappa B
NFKB1A	Nuclear Factor Kappa B Inhibitor Alpha
NPV	Negative Predictive Value
NSAID	Non-Steroidal Anti-Inflammatory Drug
PPV	Positive Predictive Value
qRT-PCR	Quantitative Real-Time Polymerase Chain Reaction
ROC	Receiver Operating Characteristic
SIRPα	Signal Regulatory Protein Alpha
*STAT1*	Signal Transducer and Activator of Transcription 1
TNF-α	Tumor Necrosis Factor Alpha
*TNFAIP3*	TNF Alpha-Induced Protein 3
TRIM	Tripartite Motif
VAS	Visual Analog Scale
